# Machine Learning on the Pediatric Intensive Care (PIC) Database: PIC-Powered Prediction

**DOI:** 10.3390/bioengineering13090978

**Published:** 2026-08-26

**Authors:** Hammad Ashraf Ganatra, Daniah Shamim, Shawn B. Sood, Amr Mohamed Ali

**Affiliations:** 1 Pediatric Critical Care Medicine, Cleveland Clinic Children’s Hospital, Cleveland, OH 44195, USA; 2 Neurological Institute, Cleveland Clinic, Cleveland, OH 44195, USA; 3 Pediatric Critical Care Medicine, University of Missouri School of Medicine, Columbia, MO 65212, USA; 4 Pediatric Cardiac Critical Care, West Virginia University Children’s, Morgantown, WV 26506, USA

**Keywords:** pediatric intensive care units, machine learning, critical care, electronic health records, forecasting, hospital mortality, sepsis, acute kidney injury

## Abstract

Machine learning (ML) applied to pediatric intensive care data could enable earlier risk stratification and more precise decision support, but limited shareable pediatric datasets have constrained progress. The Pediatric Intensive Care database (PIC) on PhysioNet provides a de-identified, bilingual electronic health record resource spanning 2010–2018. In this narrative review we synthesize ten PIC-based ML studies identified through forward citation tracking on the original PIC publication and PhysioNet dataset record in PubMed, Web of Science, and Google Scholar. For each study we abstracted the clinical question, cohort, label construction, feature engineering, model class, validation design, calibration reporting, and release of reproducibility artifacts. The reviewed studies addressed catheter-associated thrombosis, sepsis, in-hospital mortality, and organ-dysfunction phenotyping. We organize the synthesis around four substrate properties of PIC: irregular time series, constructed labels, single-center temporal drift, and bilingual identifier semantics. The review advances three claims. First, upstream design choices appear to drive performance at least as much as classifier selection across the studies reviewed. Second, within-site discrimination metrics are insufficient without calibration, decision-curve analysis, and deployment-realistic validation. Third, PIC should be viewed as the seed for a collaborative pediatric ICU data ecosystem.

## 1. Introduction

Pediatric critical care is not simply intensive care for “miniature adults.” In the pediatric world, physiologic norms shift across developmental stages, therapeutic indices are narrower, and pharmacokinetics and -dynamics diverge from adult patterns [1,2]. Outcomes are further shaped by devices, dosing logistics, and care pathways that differ fundamentally from adult practice. Nevertheless, much of the historical machine-learning (ML) literature in critical care has been trained and validated on adult datasets such as MIMIC (Medical Information Mart for Intensive Care) and eICU Collaborative Research Database or on underpowered pediatric registries [3,4,5]. The consequence is predictable: models transplanted to the Pediatric Intensive Care Unit (PICU) are often poorly calibrated, brittle under distribution shift, and difficult to adopt at the bedside [6,7].

The Pediatric Intensive Care database (PIC) on PhysioNet has helped rebalance this landscape by providing a pediatric-specific electronic health record (EHR)–derived relational database resource with sufficient breadth and longitudinal depth for methodologically credible modeling [8,9]. PIC integrates time-varying vital signs, comprehensive laboratory and medication data, procedure and diagnosis codes, and a dedicated perioperative vital-sign stream. The relational schema mirrors adult critical-care datasets (patients/admissions/ICU stays with indexed tables) while retaining pediatric-specific nuance (age/weight normalization, pediatric units and reference ranges). Records originate from a large tertiary children’s hospital and span 2010–2018 across multiple intensive care units (medical-surgical, cardiac, and neonatal), comprising approximately 12,881 unique patients and 13,944 ICU stays. The database is de-identified with per-patient date shifting that preserves within-patient temporal structure (e.g., seasonality) and is made available internationally under a data-use agreement following human-subject training [8]. Across the wider pediatric ICU machine-learning literature, investigators have leveraged these assets to address core PICU questions such as near-term and in-hospital mortality, length of stay, sepsis recognition and early detection, acute kidney injury (AKI) and other organ dysfunction, ventilatory milestones such as extubation readiness or failure, perioperative hypotension and postoperative complications, and more recently, cardiac-arrest early warning [10].

Publicly available pediatric critical care EHR datasets with sufficient longitudinal depth for ML remain scarce. Administrative registries such as VPS (Virtual PICU Systems) and PICANet (Paediatric Intensive Care Audit Network) [11,12] offer multi-center breadth but lack the time-stamped vital signs, laboratory trends, and medication records that ML feature engineering requires. PIC is currently the only open resource that combines relational EHR depth with international accessibility under a standard data-use agreement—making it the de facto substrate for reproducible PICU-based ML research and the natural focal point for a methodological review of this literature.

This narrative review synthesizes ten PIC-based ML research studies published to-date and advances three claims. First, that upstream design choices (such as label hygiene, time anchoring and prediction gaps, feature windowing for irregular time series, and principled treatment of missingness) appear to drive performance, reproducibility, and clinical utility at least as much as the choice of classifier across the studies reviewed. Second, that within-site discrimination metrics (e.g., area under the receiver operating characteristic curve (AUROC)) are insufficient without calibration assessment, decision-curve analysis, and temporal or external testing that approximates deployment. Third, that PIC should be viewed as a seed for a collaborative pediatric ICU data ecosystem built on shared phenotypes, versioned item mappings, and packaged, easy-to-share data-processing steps that make the results consistent, transparent, and clinically trustworthy. With this review, we place PIC within the larger pediatric data landscape, where there has long been a push for open, high-quality pediatric critical-care datasets to complement existing administrative registries. These resources are essential for producing reproducible machine-learning studies and trustworthy method development.

## 2. The PIC Database as a Modeling Substrate: Structure, Strengths, and Pitfalls

Before reviewing the individual studies, it is worth examining the properties of PIC itself, because several of these properties directly shape which design choices are credible and which are not. PIC’s relational schema will look familiar to investigators familiar with adult critical-care datasets: patients/admissions/ICU stays; time-stamped chart events; labs and microbiology; medications and fluids; diagnoses and procedures; and outcomes. Several features, however, require pediatric-aware ML design.

### 2.1. Irregular Multivariate Time Series

PIC reflects the rhythm of real clinical care rather than a fixed sampling schedule. Vital signs are charted asynchronously, more frequently in unstable patients and less often in stable ones. Laboratory tests appear in the record only when a clinical question prompts them, so the very presence of a lab can carry diagnostic intent. Medication timestamps reflect nursing workflow as much as biology, since a dose hung at 10:17 may be charted against the 10:00 order. Perioperative streams are dense during surgery and absent outside it. This irregularity is informative, not noise.

Modelers who flatten these patterns onto a fixed-interval grid through naïve resampling or last-value carry-forward face two specific risks. First, the trajectory shape that carries the predictive signal—the slope of a rising creatinine, the climb of a heart rate before deterioration—can be smoothed away into averaged values, eliminating the very feature the model was meant to detect. Second, information recorded after the prediction time-zero can leak backward into earlier training rows, producing optimistic in-sample performance that collapses at deployment.

### 2.2. Operational Labels and Phenotyping

PICU outcomes must be constructed through clinical phenotyping rather than retrieved from pre-labelled fields. Sepsis status, for instance, depends on consensus criteria that evolved across the study period [13,14,15], so the same patient record can be relabeled into different cohorts depending on the criteria applied. Similarly, acute kidney injury requires a Kidney Disease: Improving Global Outcomes (KDIGO)–consistent algorithm together with careful baseline creatinine estimation [16,17], since most PICU patients arrive without a documented pre-admission creatinine. The outcome label is therefore a methodological artifact in its own right, and downstream model performance depends as much on how rigorously it was constructed as on the model that consumes it.

### 2.3. Single-Center Design and Temporal Drift

PIC’s single-center origin shapes every record it contains. Local practices act as hidden confounders that a model can inadvertently learn as a “site signature” rather than as transferable physiology. The data is also non-stationary across the 2010–2018 window. A change in ventilator-weaning protocol or a coding-guideline update can move learned features even when the underlying biology has not changed. Furthermore, per-patient date shifting compounds the problem. While essential for privacy, it severs the absolute timeline that would otherwise let an investigator align records with external events such as protocol revisions or guideline releases, and it can also obscure implicit time peeking, the accidental use of information from after the prediction time. Taken together, these three forces mean that PIC-derived models should be evaluated against the possibility that they have learned the institution and the era as much as the disease.

### 2.4. Item Dictionaries and Bilingual Labels

PIC indexes its clinical events with character-typed item identifiers and bilingual Chinese-English labels, an organizational choice that places the burden of variable definition on the analyst rather than on the database. Unlike MIMIC, where the ITEMID is an integer and where a community-maintained concept layer resolves identifiers to canonical clinical concepts, PIC has no equivalent shared layer, and the database’s own authors acknowledge that language ambiguities may persist for terms without a standard code. Therefore, many common clinical concepts can potentially appear under multiple labels with subtly different wording, in either or both languages, and the burden falls on the research team to ensure consistency.

These properties make PIC a credible testbed for ML methods that respect temporal structure, label construction, site effects, and identifier semantics. But these are also the fault lines along which weak design may surface.

## 3. Methods of the Narrative Synthesis

**Figure 1:**
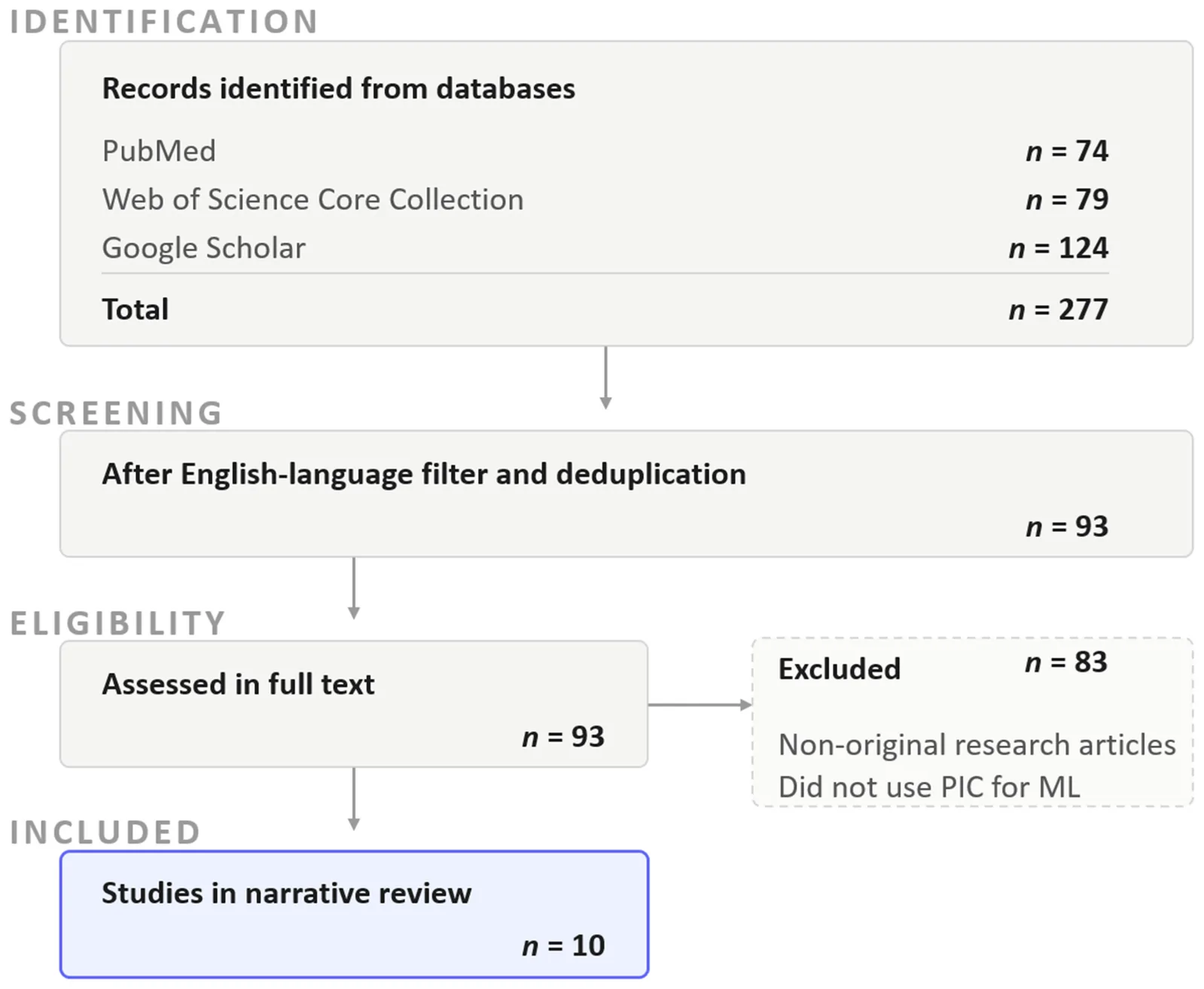
PRISMA flow diagram of the literature search and study selection.

We identified candidate articles through forward citation tracking on the original PIC database publication [8] and the PIC dataset record on PhysioNet [18]. Forward citation lists were retrieved from PubMed, Web of Science Core Collection, and Google Scholar on 27 November 2025. No search strings were entered. Records were drawn directly from the cited-by lists of the two seed items, returning 74, 79, and 124 records, respectively. The search was not updated after this date, as this review was not intended as a living or systematic review. After restricting to English-language articles and deduplicating across the three databases by DOI and bibliographic-record matching, 93 unique records remained. The lead author and one co-author independently first screened all 93 records by title and abstract and then retrieved and reviewed full texts for any record that either reviewer deemed potentially eligible. Disagreements at either stage were resolved through discussion and consensus between the two reviewers, with no cases requiring adjudication by a third party. The number of screening disagreements was small and did not affect the final set of included studies. Articles were eligible for inclusion if they (1) reported the original development or evaluation of a supervised or unsupervised machine-learning model, (2) used the PIC database as the primary or sole data source for model training or validation, and (3) were published as peer-reviewed full-text journal articles in English. We excluded non-original research (commentaries, reviews, editorials), conference abstracts, preprints, and studies using PIC only as a secondary or supplementary dataset alongside another primary database. We also excluded studies that did not use the PIC database for machine-learning model development, including those that used it solely for epidemiological analyses or traditional statistical modeling. One study that originally developed a model on MIMIC and subsequently validated it on PIC was retained, as PIC served as the direct evaluation environment. No restrictions were placed on publication date. Ten studies met these criteria and were included in this narrative review. Although this is a narrative rather than systematic review, we adopted the PRISMA flow diagram format to enhance transparency of the study selection process; this does not imply adherence to full systematic review methodology (Figure 1).

We conducted a structured quality assessment of all ten included studies across six domains that were deemed, in the authors’ judgment, to be the methodological dimensions most consequential for deployment-oriented clinical ML. “Risk of bias” captures whether model development is sound enough to trust; “validation strategy” reflects whether performance estimates generalize beyond the training context; “calibration reporting” indicates whether predicted probabilities are clinically actionable; “reproducibility artifacts” determine whether the work can be independently verified; “missing data handling” addresses a near-universal challenge in her-derived cohorts; and “clinical applicability” assesses whether the prediction task and output are structured for bedside use. Formal PROBAST scoring was not performed given the heterogeneity of clinical tasks and modeling approaches across included studies, though the six domains were selected to remain broadly aligned with its conceptual dimensions. The use of an author-defined rather than formally validated framework limits the comparability of ratings across studies and prevents direct benchmarking against the literature using standardized tools such as PROBAST. Readers should therefore interpret the domain ratings as structured expert judgments rather than scores derived from a validated instrument. Domain ratings were assigned by the lead author and reviewed by a co-author, with disagreements resolved through discussion and consensus.

For each included study, we abstracted the following:

1. **The clinical question and prediction horizon** (e.g., 6 h sepsis warning, 24 h mortality prediction, extubation-failure prediction);
2. **Cohort construction** (inclusion/exclusion criteria and handling of repeated admissions or ICU stays);
3. **Label construction and leakage-mitigation strategy** (time-zero definition, prediction gap, and removal/censoring of post-event features);
4. **Feature engineering approaches** (static vs. time-varying features, window definitions, delta features, composite indices), missing-data handling (simple vs. learned imputation, use of missingness indicators), and class-imbalance strategies;
5. **Model classes** (regularized logistic regression, gradient boosting, SVMs, sequence models, transformers) and explainability methods, where reported (global feature importance, SHapley Additive exPlanations [SHAP], partial-dependence plots);
6. **Validation design** (random vs. temporal splits, external testing, subgroup analyses), calibration methods (reliability plots, Brier scores, recalibration), and decision-analysis strategies (threshold-specific operating points, decision-curve analysis); and
7. **Public release of reproducibility artifacts, where available**, including code, item-mapping tables, and containerized preprocessing/modeling pipelines.

Because the included studies vary substantially in reporting completeness and label definitions, our synthesis focuses on design quality, methodological rigor, and reproducibility, rather than attempting any meta-analytic pooling.

## 4. Results: Thematic Synthesis of Study Methodology and Performance

The ten PIC-based studies cluster into four clinical-problem groups. For each group, a shared comparison table summarizes cohort construction, outcome definition, feature engineering, model class, validation strategy, calibration/decision-analytic reporting, and code availability. The narrative associated with each table focuses on what is methodologically distinctive or worth flagging in each study rather than recapitulating results already visible in the table. Of note, the findings below should be interpreted in light of the search strategy’s inherent limitations, which are discussed fully in the Limitations section.

### 4.1. Catheter-Associated Thrombosis (Li 2021 [19], Li 2022 [20])

Both Li studies [19,20] address central venous catheter–associated deep venous thrombosis (CADVT) in the same institutional CVC cohort window (Dec. 2015–Dec. 2018); thus, they are two analyses of the same underlying population rather than independent replications (Table 1). The distinction between them is the modeling lens: Li (2021) [19] targets actionable pre-event lead time via a multimodal temporal architecture; Li (2022) [20] targets risk-factor discovery via fusion ensembles of classical ML.

Li (2021) [19] is notable for explicitly quantifying the lead-time/discrimination trade-off—AUROC held flat from 24 to 72 h before event recognition—and for being one of the few PIC studies to report calibration via Brier and Spiegelhalter’s test, from which the authors themselves conclude the model would need recalibration before bedside use. The best single-horizon operating point was at 24 h (accuracy ≈ 0.77, recall ≈ 0.90).

Li (2022) [20] converges on a compact 11-variable risk-factor set (acid–base markers: pH, bicarbonate, standard base excess; PaO_2_; coagulation: D-dimer, thrombin time; dehydrating agents; leukocyte indices) through permutation-style accuracy drops across multiple base learners, offering some methodological triangulation. The clinical epidemiology is consistent across the two papers: CADVT patients were older, more often male, and had longer dwell time, with higher risk for intracranial space-occupying lesions, bleeding, and infection/inflammation and with longer ICU/hospital stays and higher mortality (5.2% vs. 2.4%).

### 4.2. Sepsis and Cross-Population Generalization (Nguyen 2023 [21], Marassi 2023 [22])

Two studies address sepsis from very different angles (Table 2). Nguyen [21] frames it as a probabilistic-graphical diagnostic task within a single PIC release and authors the only study in this review to report adherence to the Transparent Reporting of a multivariable prediction model for Individual Prognosis Or Diagnosis (TRIPOD) reporting checklist [23,24,25]. Marassi [22] proposes a physiology-normalization transformation (Continuous, Age-Normalized Sequential Organ Failure Assessment, CAN-SOFA) that allows sepsis models to transfer bidirectionally between PIC and MIMIC—the only cross-population transfer experiment in the literature.

Nguyen (2023) [21] turns missingness into a modeled signal by treating “no record” as an explicit fourth discretization category alongside low/normal/high. The finding that a 48 h feature window outperforms 24 h data points to additional physiologic signal accumulating in hours 24–48. The headline sensitivity of 0.46 is a clinically important ceiling—more than half of true sepsis cases missed at the default operating point—that should be read alongside the strong specificity (0.95) and negative predictive value (NPV; ≈0.97). Microbiology contributed little to sensitivity, largely because 67% of sepsis cases had negative cultures.

Marassi (2023) [22] is the only PIC study attempting cross-population transfer. The result—that an adult-trained model generalizes to pediatrics with 0.77 accuracy without retraining, and that retraining on adult data then testing on PIC increases accuracy to 0.95—is the strongest evidence in this review that disciplined physiology normalization is as important as the downstream model. The reliance on ICD-coded sepsis and ICU-transfer time-zero is an acknowledged limitation, and neither AUROC nor calibration was reported.

### 4.3. Mortality and Prognostic Prediction (Hong 2021 [26], Prithula 2024 [27], Jia 2025 [28])

Three studies built prognostic models for in-hospital mortality on different cohort filters—general PICU admissions (Hong), respiratory-disease admissions (Prithula), and pneumonia admissions (Jia) [26,27,28]. All three defined the mortality label via the ADMISSIONS table’s HOSPITAL_EXPIRE_FLAG, relied on random rather than temporal splits, and did not report calibration or decision-curve analysis (Table 3).

Hong (2021) [26] is the reference example in this group for disciplined, interpretable baselines: the 11-feature logistic model retained nearly all of the discrimination of the 397-feature version and outperformed the Pediatric Risk of Mortality, third generation (PRISM III) [29] on both AUROC and area under the precision-recall curve (AUPRC) (LR-best AUPRC 0.176 vs. PRISM III 0.140), reinforcing the value of strong baselines before pursuing model complexity. The code is publicly available at github.com/hsd1503/PIC_mortality (accessed on 21 August 2026).

Prithula (2024) [27] contributes a technical innovation—partitioning the majority class and applying the Synthetic Minority Over-sampling Technique (SMOTE) within each subset—that substantially improved minority-class sensitivity when paired with CatBoost. The design-level gap is the absence of any defined time-zero or prediction window, which leaves the leakage profile unquantified.

Jia (2025) [28] combines four feature-selection techniques (stepwise regression, Pearson/Spearman correlation, random-forest importance, and grey relational analysis) and screens 113 algorithmic combinations (the paper’s Figure 1 reports 101—a minor reporting discrepancy). The ~0.19 gap between training and test AUROC is a strong overfitting signal the paper’s own framing does not fully reckon with, and the 543-patient cohort is the smallest in this review.

### 4.4. Organ-Dysfunction Phenotyping (Zeng 2023 [30], Shen 2025 [31], Zhou 2025 [32])

Three studies build phenotype-driven models for specific organ-dysfunction syndromes—postoperative AKI after pediatric cardiac surgery (Zeng), mortality among PICU patients with AKI (Shen), and disseminated intravascular coagulation (Zhou) [30,31,32]. All three invest heavily in labeling rigor and feature-selection discipline relative to the rest of the literature and together account for every explicit calibration or decision-curve report in this review (Table 4).

Zeng (2023) [30] uses an unusual three-way split (train/calibration/test) specifically engineered to support post hoc recalibration; this is the cleanest example in this review of a pipeline designed end to end for deployment. SHAP interpretation surfaced clinically actionable signals: autologous blood transfusion during cardiopulmonary bypass (CPB) was protective, while prolonged mechanical ventilation was risk-increasing. The pre-onset AUROC curve quantifies the lead-time/performance trade-off directly.

Shen (2025) [31] pairs the ML model with Kaplan–Meier and restricted cubic spline analyses, which test rather than assume the shape of the lactate–mortality association; the RCS confirmed a largely linear relationship above 1.5 mmol/L, with effects most pronounced in infants (0–3 years) and AKI stage 1 patients. Lactate consistently ranked as the top SHAP predictor across all three horizons.

Zhou (2025) [32] demonstrates the most disciplined first-mile of any study in the review, using variance inflation factor (VIF) to rule out multicollinearity before feature inclusion, Akaike Information Criterion (AIC)–guided stepwise selection, multiple imputation by chained equations (MICE) [33] for missingness, and decision-curve analysis [34,35] to quantify net clinical benefit at different operating thresholds. Random forest achieved the highest raw accuracy but only moderate Kappa (0.472), illustrating why the authors selected extreme gradient boosting (XGB) on the basis of overall discrimination and net benefit rather than accuracy alone. The neonatal exclusion is consequential for the study’s applicability range.

## 5. Methodological Lessons from PIC-Based Machine-Learning Studies

The following observations are drawn from the ten studies in this corpus and are intended as evidence-informed recommendations rather than universal principles, since a larger and more architecturally diverse evidence base would be needed to establish them conclusively. Where a recommendation is grounded in specific patterns observed across the reviewed studies, the relevant study or studies are cited. Where it reflects a methodological gap uniformly observed across the corpus—or a best practice from the broader clinical ML literature that none of the reviewed studies achieved—this is stated explicitly. Across these studies, calibration was formally reported in three, code was publicly released in two, and none used a temporal validation split. Sample sizes ranged from 543 to 13,258 patients, and AUROC values, where reported on held-out test sets, ranged from 0.70 to 0.91. These patterns motivate the methodological lessons that follow.

**Figure 2:**
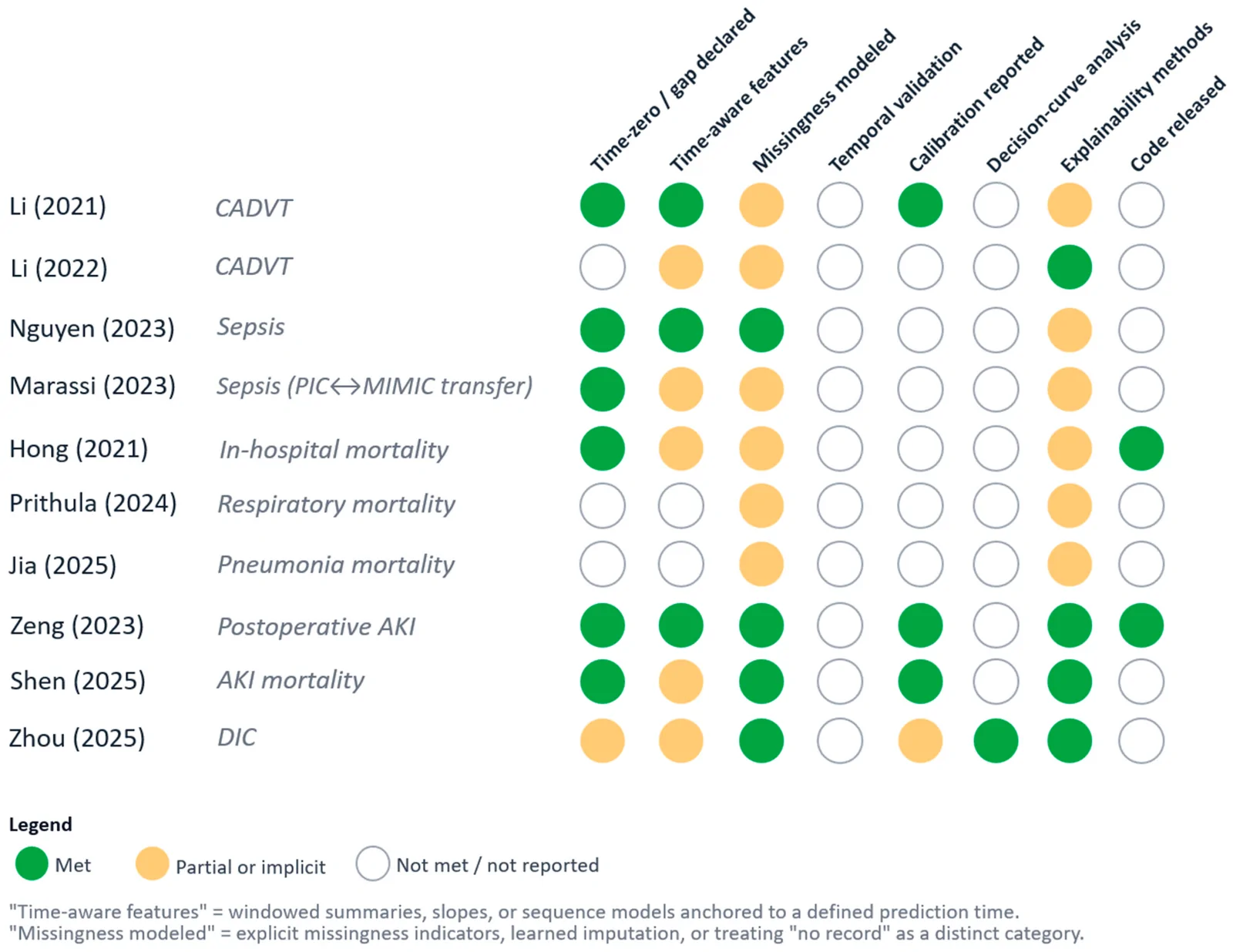
Methodological discipline across the ten reviewed PIC-based machine-learning studies [19,20,21,22,26,27,28,30,31,32].

Across the ten PIC-based investigations, a small set of recurring methodological choices largely determines whether models are merely publishable or plausibly deployable. These choices concern problem definition, time-aware feature construction from irregular data, and evaluation under deployment-relevant conditions. Figure 2 provides a cross-cutting summary of methodological discipline across all ten studies on eight evaluation criteria. Table 5 presents a formal structured quality assessment of the same studies across six domains—risk of bias, validation strategy, calibration reporting, reproducibility artifacts, missing data handling, and clinical applicability—and serves as the basis for the methodological conclusions drawn throughout this section.

### 5.1. Label Discipline, Not Algorithm Shopping

A consistent marker of study quality is whether time-zero and the prediction gap are explicitly declared, defended, and enforced—especially for operational outcomes such as sepsis, AKI, and extubation failure. Studies that censor post-event features, enforce timestamp monotonicity, and document exclusion windows avoid many hidden leakage vectors. When labels are partially driven by local practice (e.g., blood culture ordering, antibiotic initiation), authors should provide phenotype code and conduct sensitivity analyses (for example, definitions that exclude antibiotic features) to demonstrate that performance is not driven by workflow proxies.

### 5.2. Temporal Features Beat “Forcing the Data into a Grid”

Instead of resampling every lab/vital to fixed hourly bins, a generally more reliable approach is to summarize clinically meaningful windows: the most recent value, the min/max, the average, and whether the value is rising or falling (slope), ideally adjusted for age/weight. Simple physiologic summaries (e.g., shock index [36,37,38] or cumulative vasoactive exposure) can also carry a strong signal. Deep learning on raw time series tends to help only when the sequence is aligned to a real decision point (with a clear prediction gap) and when it clearly outperforms well-calibrated baseline models; otherwise, it mostly re-learns what these windowed summaries already captured.

### 5.3. Missingness Is Informative and Warrants Explicit Modeling

In pediatric intensive care, laboratory ordering is selective and often reflects evolving clinical hypotheses. Treating missingness as signal—by including missingness indicators alongside principled imputation—typically improves discrimination and calibration compared with mean imputation or complete-case analysis [39,40,41]. Reporting the missingness profile by variable and by window (before and after feature construction) should be standard, since performance can be driven as much by ordering behavior as by physiology.

### 5.4. Validation Must Resemble Deployment

The key question is: will the model still work next year or in another ICU? Random “train/test” splits can look better than reality because they mix old and new practice patterns. A more honest test is a temporal split (train on earlier years, test on later years) and, ideally, external validation at a different hospital. If external data are unavailable, use a distant time period as a stress test and report how calibration drifts over time, along with a simple plan for periodic recalibration. It is worth stating plainly that none of the ten studies reviewed here used a temporal validation split, which is arguably a consequential cross-cutting methodological gap in this literature and one that future PIC-based modeling effort should make a priority to address.

### 5.5. Calibration and Decision Utility, Not AUROC Alone

AUROC (and AUPRC, especially when events are rare) reflects discrimination—how well the model separates higher- from lower-risk patients. It does not tell you whether the risk estimate is accurate (calibration) or whether using the model would improve decisions. Studies should show calibration (e.g., do patients predicted at ~10% risk have ~10% event rates?) and report thresholded, workflow-relevant performance (e.g., at ~5 alerts/day, what fraction are true positives and what is the sensitivity?).

### 5.6. Transparency of the “First Mile”

The hardest (and easiest to get wrong) step is how you pull and define the data. In PIC, the same clinical concept (e.g., “creatinine”) may appear under different local codes/names and units. A shared “mapping table” that lists exactly which PIC items were used for each variable, and how units were handled, lets other teams reproduce the same dataset. Likewise, sharing the label (phenotype) code and feature-extraction scripts (with versioning) is strongly recommended; without them, models are difficult to reliably compare or replicate. Together, these item-mapping tables, phenotype code, and feature-extraction scripts constitute what this review terms “first-mile artifacts,” meaning the components that allow independent teams to reconstruct the same dataset from raw EHR data.

### 5.7. Emerging Architectures and Future Directions

None of the ten studies reviewed here employ transformer-based architectures, foundation models, or large language model approaches, with the literature drawing predominantly from classical machine learning, ensemble methods, and recurrent neural networks. This partly reflects a genuine property of PIC as a substrate: the database is primarily structured and tabular, with a limited free-text clinical narrative, which constrains the direct applicability of language model architectures that depend on rich unstructured text. Transformer architectures applied to structured EHR time-series data represent a tractable near-term direction for PIC-based research. Multimodal transformer approaches have already been applied in adjacent pediatric critical care contexts, such as cardiac arrest prediction, demonstrating that the field is moving in this direction [42]. Emerging EHR foundation models that tokenize structured clinical events rather than text may eventually be applicable to PIC as the field matures. Multimodal architectures integrating structured and unstructured data streams remain a longer-term aspiration, contingent on enrichment of PIC with free-text or imaging data beyond its current scope. A comprehensive treatment of emerging architectures and their implications for pediatric critical care ML is available in the broader literature [10,43]. Broader deployment considerations, including regulatory approval pathways, post-deployment monitoring, and governance frameworks for clinical ML tools, remain important practical challenges that fall outside the methodological scope of this review and are addressed in the wider implementation science literature.

Table 6 provides a concise cross-cutting summary of the principal methodological gaps identified across the ten studies and the corresponding recommendations for future PIC-based research.

## 6. How Robust Is the Pediatric Intensive Care (PIC) Database for Machine Learning? Strengths, Limitations, and Realistic Expectations

PIC is unusually useful for pediatric critical-care ML because it combines broad clinical coverage with longitudinal structure. Across the ten PIC-based studies reviewed, investigators were able to build models from routinely available domains—admissions/ICU stays, demographics, diagnoses/procedures, medications/fluids, laboratories, and time-stamped vital signs—without requiring bespoke data pulls.

Several studies explicitly leveraged the longitudinal nature of PIC to move beyond static “first-day” snapshots. For example, sepsis-focused work constructed short-horizon prediction windows and demonstrated that time-aware features (e.g., trends in lactate, evolving hemodynamics, and escalating vasoactive exposure) can be informative when anchored to a clear prediction time-zero.

PIC also offers at least one distinctive technical advantage: a dedicated peri-operative vital-sign stream (e.g., SURGERY_VITAL_SIGNS) sampled at higher frequency than routine chart-event tables. This stream supports intraoperative and postoperative risk modeling using higher-frequency intraoperative vitals, a capability often absent or harder to reproduce in open adult critical-care datasets. Among the ten studies reviewed, only Zeng (2023) [30] exploits this resource, resampling at 5 min intervals to support time-aware AKI prediction after cardiac surgery. The stream therefore represents an under-utilized asset of PIC that future investigators studying surgical or peri-operative outcomes could leverage. In aggregate, the ten studies show that PIC can support both strong classical baselines (regularized regression, gradient boosting) and recurrent architectures, provided that investigators define prediction horizons, time anchoring, and feature windows carefully.

PIC’s limitations are not unique, but they are particularly consequential for pediatric ML. PIC is single-center. Practice patterns—lab-ordering thresholds, antimicrobial choices, vasopressor titration habits, ventilator-weaning culture—can be learned by models as hidden “site signatures.” This risk is most obvious in tasks where the label is partly operational. For sepsis, for instance, phenotypes that rely on blood-culture timing and antibiotic initiation can inadvertently train a model to detect workflow rather than physiology. In the reviewed sepsis work, careful prediction gaps and feature censoring reduce this risk, but the underlying dependence on local practice remains a central barrier to portability. A related concern is patient selection bias. PIC’s source institution is a large tertiary referral center whose case-mix and admission thresholds may differ substantially from those of community hospitals or smaller pediatric centers, meaning models trained on this population may encode selection effects that limit applicability even within the same healthcare system.

The demographic composition of the PIC cohort also warrants acknowledgment. The patient population reflects the catchment area of one institution, and none of the ten reviewed studies reported subgroup analyses by age group, sex, or comorbidity profile, limiting the ability to assess whether model performance is consistent across the demographic subgroups most relevant to pediatric critical care. The absence of subgroup analyses also means that differential model performance across age bands, sex, or clinical severity cannot be assessed, raising unresolved questions about algorithmic fairness in this literature.

The generalizability of PIC-based models is best understood as a cross-institutional portability question rather than a geographic one. The same structural barriers apply whether the target institution is in China, North America, or Europe. Marassi (2023) [22] authors the only study in this review to test cross-institutional transfer, and its finding that disciplined physiologic normalization substantially reduced the performance gap suggests this barrier is addressable through principled feature design.

It is worth situating PIC within the broader landscape of available critical care databases to distinguish which of its challenges are database-specific and which are universal to the field. Open, EHR-derived databases suitable for pediatric ICU machine learning remain scarce. The most structurally comparable resource is MIMIC, which is adult-focused but shares a relational schema, time-stamped clinical events, and single-center origin with PIC. Administrative registries such as VPS and PICANet offer broader multi-center coverage but lack the longitudinal EHR depth required for most ML modeling tasks. Several of PIC’s challenges are universal to single-center EHR databases and are well-documented in the MIMIC-based ML literature, including temporal drift, workflow-driven labels, and generalizability limits. Others are PIC-specific: the bilingual identifier scheme, Chinese institutional practice patterns, per-patient date shifting that severs the absolute timeline, and the Sepsis-3 definitional transition within the 2010–2018 study window are features of this database that investigators working with other resources would not encounter. Recognizing this distinction helps readers calibrate which methodological lessons from this review are transferable to pediatric critical care ML broadly and which are specific to the PIC substrate.

Non-stationarity across the 2010–2018 study window is expected. Protocol updates, documentation changes, and case-mix shifts can degrade model calibration over time, even when discrimination appears stable. None of the ten reviewed studies addressed this through a temporal validation split (training on earlier years and testing on later years), and none reported year-by-year drift or specified a recalibration plan [6,44,45]. Marassi’s study (2023) [22] is the only one to attempt a non-random generalization test, but its cross-population transfer between PIC and MIMIC addresses pediatric-versus-adult drift rather than within-PIC temporal drift. For a clinician reader, the implication is simple: even a high-performing PIC model should be assumed to require periodic recalibration if deployed, and the existing literature provides no empirical basis for estimating how quickly that recalibration becomes necessary.

The 2010–2018 study window straddles a major transition in sepsis definitions: Sepsis-3 was published in 2016, and the first pediatric-specific consensus criteria was not published until 2024. Studies in this review that address sepsis therefore applied different definitional frameworks, and apparent differences in model performance may partly reflect this heterogeneity rather than modeling choices alone. This concern is directly compounded by PIC’s per-patient date shifting: because absolute timestamps are randomized during de-identification, investigators cannot determine whether any given admission occurred before or after Sepsis-3′s publication, making it impossible to control for definitional evolution or to align records with the institutional protocol changes that likely followed it.

Harmonization requires deliberate, manual standardization. Beyond the bilingual identifier scheme described in Section 2, the same lab assay can be reported in different units (e.g., creatinine in μmol/L vs. mg/dL), and without an explicit unit-handling table, this fragmentation is silent. This is a reproducibility risk rather than a performance risk, and it can quietly undermine cross-paper synthesis.

In practice, PIC-based models should be viewed as strong candidates for benchmarking and hypothesis generation, but they typically require re-validation and often recalibration before use in other hospitals. Any institution considering deployment should account for three practical barriers. First, temporal drift from protocol and case-mix changes can silently degrade calibration over time. Second, workflow-driven labels risk training models to detect institutional practice patterns rather than underlying physiology. Third, documentation heterogeneity, including bilingual identifiers and unit variation across assays, can complicate feature mapping at the receiving site.

## 7. Practical Takeaways for Clinicians

The methodological lessons in Section 5 and the strengths-and-limitations discussion in Section 6 translate into different practical guidance, depending on a clinician’s relationship to PIC-based work. The two boxes below distill that guidance for the two most common clinical audiences: clinicians reading published PIC-based ML papers (Box 1) and clinicians using PIC for their own research (Box 2).

Box 1 **Practical takeaways for clinicians reading PIC-based ML papers.**

- Trust models that state a clear prediction horizon and show a “prediction gap” (features stop before the event).
- Prefer studies that test on later time periods (temporal split) and report calibration, not just AUROC.
- Be cautious when the outcome depends on workflow (e.g., cultures/antibiotics), because models may learn practice patterns.
- Look for transparency: a published item “translation table” and phenotype/feature code makes results reproducible.
- Expect recalibration if a model is moved to a different PICU or applied years later.

Box 2 **Practical takeaways for clinicians using PIC for their own research.**

- Pin down the clinical decision and time-zero before touching the data and prevent post-event features from leaking into training.
- Engineer time-aware features (last value, min, max, slope, deltas, composites like shock index) rather than resampling to a fixed grid.
- Treat missingness as informative—add missingness indicators alongside imputation and report the pattern by variable and window.
- Start with strong, interpretable baselines (regularized logistic regression, calibrated gradient boosting); escalate to deep models only if they clearly outperform under temporal validation.
- Validate on time you did not train on; when external data are unavailable, stress-test with distant temporal slices, subcohort holds, and shift perturbations.
- Report calibration (reliability plot, Brier score) and decision utility (decision curves at clinical alert budgets), not AUROC alone.
- Release your first-mile artifacts—item-mapping table, phenotype code, feature scripts—so others can reproduce your cohort exactly.

## 8. Limitations of This Review

Candidate articles were identified through forward citation tracking on the original PIC database publication and the PhysioNet dataset record. This approach was chosen because most methodologically credible peer-reviewed studies using PIC would be expected to formally cite these seed items, providing high precision for this database-specific review. Keyword-based searching was not performed, as terms broad enough to capture all PIC-based work would return hundreds of thousands of results spanning the full breadth of pediatric critical care, making the scope of this review operationally and methodologically unworkable. Conference abstracts and preprints were deliberately excluded rather than missed, as our inclusion criteria required peer-reviewed full-text journal articles. Nonetheless, this approach carries structural limits. The most significant is the potential for Chinese-language publications in databases such as CNKI or Wanfang, which would not appear in forward citation lists on PubMed, Web of Science, or Google Scholar. Given that PIC originates from a Chinese institution, such publications may exist, and their number cannot be precisely quantified. Similarly, studies that referenced PIC informally without a formal citation to the database descriptor paper or dataset DOI would also fall outside the search. This could include studies that linked only to the PhysioNet repository page rather than the peer-reviewed descriptor, studies that cited a downstream PIC-based paper in lieu of the original seed publication, or studies published after a potential versioning or re-release of the database that generated new citable identifiers not captured by the two seed items. We consider these gaps unlikely to include high-impact peer-reviewed work but acknowledge them as an inherent limitation of forward citation tracking and a priority for any future update of this literature. We also acknowledge that restricting inclusion to peer-reviewed journal articles may introduce publication bias, as studies with null or modest findings are less likely to reach full peer-reviewed publication.

The included literature itself imposes further constraints. Heterogeneity across the ten studies in label definitions, preprocessing transparency, validation choices, and reporting completeness precludes both quantitative meta-analysis and formal PROBAST-style risk-of-bias scoring of individual studies [46,47]. Across the ten studies, only one released any code (Hong 2021 [26]), and none released item-mapping tables or containerized preprocessing pipelines, which limits our ability to verify replication claims or to attribute performance differences to modeling choices rather than to preprocessing. Two structural features of the corpus deserve explicit emphasis. First, none of the ten reviewed studies used a temporal validation split, so reported discrimination should be read as a within-period estimate rather than a deployment-realistic one [6,44,45]. Second, because all studies draw from the same single-center PIC database, patient-level overlap is substantial in several cases. Li (2021) and Li (2022) share near-identical patient pools, both restricted to CVC-placed patients over the same 2015–2018 window, with the former likely a strict subset of the latter [19,20]. Hong (2021), Marassi (2023), Nguyen (2023), Zhou (2025), and Shen (2025) all draw from the general PICU population, with varying but overlapping date ranges, such that concordant findings across these studies do not constitute independent replication in the traditional sense [21,22,26,31,32]. Prithula (2024) and Jia (2025) similarly share a respiratory disease cohort, with Jia (2025) representing a diagnosis-specific subset [27,28]. Only Zeng’s study (2023), which is restricted to patients undergoing congenital heart surgery with cardiopulmonary bypass, can be considered a genuinely non-overlapping cohort [30]. Readers should bear this structure in mind when interpreting apparent convergence in findings across studies. A further structural feature of the corpus is the absence of any reviewed study addressing extubation failure, ventilator weaning, perioperative hypotension, or cardiac arrest, even though these are repeatedly cited as high-value pediatric ICU prediction tasks; this leaves an important class of outcomes unaddressed by the current PIC literature. We therefore emphasize design quality and reproducibility signals rather than absolute metrics.

## 9. Conclusions

The modern PIC-based ML literature demonstrates that pediatric-specific EHR data can support credible predictive modeling for clinically meaningful tasks—in-hospital mortality, sepsis, organ dysfunction (including post-operative AKI and DIC), and catheter-associated thrombosis. The work that will inspire the most confidence going forward will not be characterized by architectural complexity alone; it will also accompanied by methodological discipline—disciplined labels, time-aware feature engineering, temporal validation, calibration, decision-curve analysis, and transparent first-mile code—a combination not yet achieved by any single reviewed study. PIC’s value is twofold: it enables credible pediatric ML today and provides the practical substrate to build a collaborative pediatric ICU data ecosystem tomorrow. Several priorities follow from this review. Reproducibility requires shared item-mapping dictionaries, released phenotype code, and containerized preprocessing pipelines so that cohorts can be reconstructed and analyses re-run. Beyond reproducibility infrastructure, temporal validation merits routine consideration in future PIC-based modeling efforts, and reporting must pair calibration and decision utility with discrimination metrics. This is how PIC-based ML can move from internally validated benchmarks toward tools that meaningfully support pediatric critical care.

## Figures and Tables

**Table 1:** Studies applying machine learning for catheter-associated complications.

Study	Cohort	Outcome and Time Anchor	Feature Engineering	Model	Validation	Calibration/DCA	Headline	Code
Li 2021 [19]	Children with CVC ≥3 d (*n* = 1830; ~15% CADVT)	CADVT at 24/48/72 h pre-recognition	Statics + 12 h windowed vitals/labs + raw time series; SMOTE	CNN + LSTM (temporal) + GRU (dynamic) + FNN (static), two-stage fusion	5-fold CV	Brier + Spiegelhalter reported; model poorly calibrated, needs recalibration	AUROC 0.83/0.82/0.82 at 24/48/72 h	Not released
Li 2022 [20]	Same CVC cohort (*n* = 3871; 9.85% CADVT)	CADVT (no lead-time framing)	478 → 74 → 11 via permutation importance across models; SMOTE	Stacking/blending (LR, RF, GBDT base learners)	5-fold CV	Not reported	Stacking AUROC 0.82; recall 0.85	Not released

*Abbreviations: AUROC, area under the receiver operating characteristic curve; CADVT, central venous catheter–associated deep venous thrombosis; CNN, convolutional neural network; CV, cross-validation; CVC, central venous catheter; DCA, decision curve analysis; FNN, feedforward neural network; GBDT, gradient-boosted decision tree; GRU, gated recurrent unit; LR, logistic regression; LSTM, long short-term memory; RF, random forest; SMOTE, Synthetic Minority Over-sampling Technique.*

**Table 2:** Studies applying machine learning for sepsis prediction.

Study	Cohort	Outcome and Time Anchor	Feature Engineering	Model	Validation	Calibration/DCA	Headline	Code
Nguyen 2023 [21]	PIC 2010–2019, *n* = 3014 (4.4% sepsis)	Sepsis within first 24 h; dual ICD-10 OR SIRS + culture/antibiotic rule	4 variable groups → 15 combinations; PIC-cutoff discretization with “no record” as distinct 4th category	Tree-Augmented Naïve Bayes (vs. LR)	10-fold CV; TRIPOD adherence	Not reported	AUROC 0.87 (TAN); sensitivity 0.46	Not released
Marassi 2023 [22]	PIC (*n* = 12,749) + MIMIC adults	ICD-coded sepsis; ICU transfer time as time-zero proxy	11 variables converted to continuous age-aware CAN-SOFA “deviance” scores	XGBoost	Cross-population transfer (PIC ↔ MIMIC)	Not reported	Acc 0.84 on PIC; 0.95 for train-MIMIC/test-PIC	Not released

*Abbreviations: AUROC, area under the receiver operating characteristic curve; CAN-SOFA, Continuous, Age-Normalized Sequential Organ Failure Assessment; CV, cross-validation; DCA, decision curve analysis; ICD-10, International Classification of Diseases, 10th revision; LR, logistic regression; MIMIC, Medical Information Mart for Intensive Care; PIC, Pediatric Intensive Care database; SIRS, systemic inflammatory response syndrome; TAN, Tree-Augmented Naïve Bayes; TRIPOD, Transparent Reporting of a multivariable prediction model for Individual Prognosis Or Diagnosis.*

**Table 3:** Studies applying machine learning for mortality prediction.

Study	Cohort	Outcome and Time Anchor	Feature Engineering	Model	Validation	Calibration/DCA	Headline	Code
Hong 2021 [26]	All PICU admits; excl. deaths <24 h	In-hospital mortality; features from first 24 h	397 hand-crafted → 11/22/59 via ensemble stepwise selection; median imputation; inverse-frequency weighting	Logistic regression (GB used only for ranking)	80/20 random	Not reported	AUROC 0.789 (59-feat); beats PRISM III	Public
Prithula 2024 [27]	Respiratory-disease admits (*n* = 1188; ~9.5% mortality)	In-hospital mortality; no time-zero or prediction gap defined	105 → 16 via RF/ExtraTrees/XGB ranking; subset-SMOTE	CatBoost (+ 10 comparators, stacking)	Random CV	Not reported	AUROC 0.85 (CatBoost + subset-SMOTE)	Not released
Jia 2025 [28]	Pneumonia admits (*n* = 543)	In-hospital mortality; no time anchor	70 labs → 14 via stepwise + Pearson/Spearman + RF + GRA; regression-based imputation	113 algorithm combinations; best Stepglm [both] + GBM	Random split	Not reported	AUROC 0.890 train/0.698 test	Not released

*Abbreviations: AUPRC, area under the precision-recall curve; AUROC, area under the receiver operating characteristic curve; CatBoost, categorical boosting; CV, cross-validation; DCA, decision curve analysis; GB, gradient boosting; GBM, gradient boosting machine; GRA, grey relational analysis; LR, logistic regression; PRISM III, Pediatric Risk of Mortality, third generation; RF, random forest; SMOTE, Synthetic Minority Over-sampling Technique; Stepglm [both], stepwise generalized linear model (both directions); XGB/XGBoost, extreme gradient boosting.*

**Table 4:** Studies applying machine learning for predicting organ dysfunction.

Study	Cohort	Outcome and Time Anchor	Feature Engineering	Model	Validation	Calibration/DCA	Headline	Code
Zeng 2023 [30]	Post-CPB cardiac surgery (*n* = 3386)	KDIGO AKI; three lead-time perspectives	Statics + peri-op time series (5 min intra-op, 8 h post-op); sample-and-hold; SMOTE	Time-aware attention RNN (LSTM + key–query attention)	60/15/25 random (train/calibration/test)	Platt or isotonic recalibration; test-set Brier ≈ 0.061 after, 0.137 before	AUROC 0.908 at 24 h post-op; 0.862 → 0.902 from 168 h → 6 h pre-onset	Public
Shen 2025 [31]	AKI by KDIGO (*n* = 3624; no neonates)	7-/14-/28-day mortality; features from first 24 h	Boruta → 29/38/44 vars; MICE (5 × 10); SMOTE	CatBoost (+8 comparators); K–M + restricted cubic splines	Random split	Brier reported (0.052 for 28 d CatBoost)	AUROC 0.871/0.871/0.867 across horizons	Not released
Zhou 2025 [32]	Admits excl. <24 h, neonates, congenital coagulopathy (*n* = 6093; 11.2% DIC)	ISTH-defined DIC	28 → 16 via AIC stepwise; MICE; VIF check for multicollinearity	XGBoost (“Alfalfa-PICU-DIC”) + 5 comparators	70/30 random + 5-fold CV	Decision-curve analysis reported; reliability plot not reported	AUROC 0.908	Not released

*Abbreviations: AIC, Akaike Information Criterion; AKI, acute kidney injury; AUROC, area under the receiver operating characteristic curve; CatBoost, categorical boosting; CPB, cardiopulmonary bypass; Brier, Brier score; CV, cross-validation; DCA, decision curve analysis; DIC, disseminated intravascular coagulation; ISTH, International Society on Thrombosis and Haemostasis; K–M, Kaplan–Meier; KDIGO, Kidney Disease: Improving Global Outcomes; LSTM, long short-term memory; MICE, multiple imputation by chained equations; RCS, restricted cubic spline; RNN, recurrent neural network; SMOTE, Synthetic Minority Over-sampling Technique; VIF, variance inflation factor; XGB/XGBoost, extreme gradient boosting.*

**Table 5:** Structured quality assessment of the ten included studies across six methodological domains.

Study	Risk of Bias	Validation Strategy	Calibration Reporting	Reproducibility Artifacts	Missing Data Handling	Clinical Applicability
Li (2021) [19] CADVT	**Moderate** Time-zero declared; 5-fold CV only; poor calibration reported	**Partial** 5-fold CV; no temporal or external validation	**Reported** Brier and Spiegelhalter reported; poorly calibrated	**Not released** No code or item mappings available	**Partial** SMOTE applied; missingness handling not explicitly described	**High** Explicit 24/48/72 h lead-times enable prophylactic action
Li (2022) [20] CADVT	**High** No lead-time framing or time-zero declared; no calibration	**Partial** 5-fold CV only; no temporal or external validation	**Not reported** Calibration not reported	**Not released** No code or item mappings available	**Partial** SMOTE applied; missingness handling not explicitly described	**Low** No prediction horizon defined; not deployable without one
Nguyen (2023) [21] Sepsis	**Moderate** TRIPOD adherence; reasonable label; 10-fold CV; modest event count	**Partial** 10-fold CV; TRIPOD adherence; no temporal split	**Not reported** Calibration not reported	**Not released** No code or item mappings available	**Adequate** Missingness treated as explicit signal (4th category)	**Moderate** Clinically relevant; sensitivity ceiling of 0.46 limits deployment
Marassi (2023) [22] Sepsis transfer	**Moderate** Cross-population validation strength; imprecise time-zero; no AUROC	**Adequate** Cross-population transfer (PIC to MIMIC and reverse)	**Not reported** Neither AUROC nor calibration reported	**Not released** No code or item mappings available	**Not reported** Missingness handling not described	**Moderate** Proof-of-concept for transfer; not yet a deployable tool
Hong (2021) [26] Mortality	**Moderate** Clean label; interpretable model; code released; random split only	**Partial** 80/20 random split; no temporal validation	**Not reported** Calibration not reported	**Adequate** Code publicly available (GitHub)	**Partial** Median imputation applied; no missingness indicators	**High** Interpretable 11-feature model; outperforms PRISM III
Prithula (2024) [27] Mortality	**High** No time-zero or prediction gap declared; small cohort; no calibration	**Partial** Random CV only; no time-zero defined	**Not reported** Calibration not reported	**Not released** No code or item mappings available	**Partial** Subset-SMOTE applied; missingness not modeled as signal	**Low** No prediction window defined; cannot map to a clinical decision point
Jia (2025) [28] Mortality	**High** No time anchor; 0.19 train-test AUROC gap; smallest cohort in review	**Partial** Random split only; large train-test performance gap indicates overfitting	**Not reported** Calibration not reported	**Not released** No code or item mappings available	**Partial** Regression-based imputation; missingness not modeled as signal	**Low** No time anchor; overfitting signal; cohort too small for confident deployment
Zeng (2023) [30] AKI	**Low** Best label discipline; three-way split with recalibration; code released	**Adequate** Three-way split (train/calibration/test); post hoc recalibration	**Reported** Brier score reported before and after recalibration	**Adequate** Code publicly available (GitHub)	**Adequate** Sample-and-hold for irregular time series; explicit handling	**High** Specific surgical context; actionable window; DCA and SHAP reported
Shen (2025) [31] AKI mortality	**Moderate** Rigorous imputation and feature selection; random split only	**Partial** Random split only; no temporal or external validation	**Reported** Brier score reported (0.052 for 28-day CatBoost)	**Not released** No code or item mappings available	**Adequate** MICE (5 × 10 imputations); Boruta feature selection	**Moderate** Prognostically useful with SHAP; random split limits deployment confidence
Zhou (2025) [32] DIC	**Moderate** Most disciplined first-mile; DCA reported; random split only	**Partial** 70/30 random split plus 5-fold CV; no temporal validation	**Partial** DCA reported; calibration reliability plot not reported	**Not released** No code or item mappings available	**Adequate** MICE imputation; VIF check for multicollinearity	**Moderate** DCA quantifies net benefit; neonatal exclusion limits applicability

Risk of bias: Low = clean label, adequate validation, no overfitting signal, calibration reported; Moderate = one domain with limitations; High = two or more domains with significant limitations. Validation strategy: Adequate = temporal or external validation performed; Partial = random cross-validation only. Calibration reporting: Reported = calibration metric presented; Partial = decision-curve analysis only; Not reported = no calibration metric. Reproducibility artifacts: Adequate = code publicly available; Not released = no code or mappings shared. Missing data handling: Adequate = principled imputation with missingness modeled as signal; Partial = imputation applied without missingness indicators; Not reported = not described. Clinical applicability: High = actionable prediction horizon with interpretable output; Moderate = clinically relevant question with deployment limitations; Low = no prediction horizon or significant overfitting.

**Table 6:** Principal methodological gaps identified across ten PIC-based machine learning studies and corresponding recommendations for future research.

Methodological Gap	Observed Across Corpus	Recommendation for Future Work
No temporal validation splits	None of the ten studies used a temporal train/test split	Train on earlier years and test on later years as a standard validation design; report calibration drift across time periods
Calibration rarely reported	3 of 10 studies formally reported calibration	Report Brier score and reliability plots alongside AUROC as a minimum standard
Reproducibility artifacts absent	2 of 10 studies released any code; none released item-mapping tables	Release item-mapping tables, phenotype code, and containerized preprocessing pipelines with each publication
Subgroup analyses absent	None of the ten studies reported performance by demographic subgroup	Report performance stratified by age group, sex, and comorbidity profile to surface potential algorithmic fairness concerns
Label construction underspecified	The majority of studies did not explicitly declare time-zero or prediction gap	Declare time-zero, prediction gap, and leakage-mitigation strategy explicitly in the Methods section
Decision-curve analysis rare	A minority of studies reported decision-curve analysis or threshold-specific operating points	Pair discrimination metrics with decision-curve analysis and report clinically relevant threshold performance
Cohort overlap unacknowledged	Several study clusters share overlapping patient populations from the same PIC window	Disclose cohort relationships with prior PIC studies; do not treat convergent findings across overlapping cohorts as independent replication
Single-center generalizability untested	Only Marassi (2023) [22] attempted cross-institutional transfer	Report calibration drift across time periods as a minimum; pursue external validation where feasible

## Data Availability

The original data presented in the study are openly available in PhysioNet at URL: https://physionet.org/content/picdb/1.1.0/ (accessed on 20 August 2026) or DOI: 10.13026/32x9-wv38.
